# Atypically distributed cutaneous lesions of Norwegian scabies in an HIV-positive man in South India: a case report

**DOI:** 10.1186/1752-1947-2-82

**Published:** 2008-03-14

**Authors:** Vignesh Ramachandran, Esaki Muthu Shankar, Bella Devaleenal, Balakrishnan Pachamuthu, Shieh Mark Thousen, Ramalingam Sekar, Solomon Suniti, Kumarasamy Nagalingeswaran

**Affiliations:** 1 Infectious Diseases Laboratory, YRG Centre for AIDS Research and Education, VHS Campus, Rajiv Gandhi Salai, Taramani, Chennai 600 113, India

## Abstract

**Introduction:**

Immune-compromised subjects, especially those with underlying HIV disease, are prone to be infected with Norwegian scabies, where the cutaneous lesions are classically distributed over the extremities.

**Case presentation:**

We report the case of an HIV-positive 16-year-old man with severe crusted Norwegian scabies initially misdiagnosed as a dermal fungal infection. The patient had extensive, generalized, thick, hyperkeratotic, crusting, yellowish papule lesions distributed on the entire body from his scalp to his toes.

The patient was started with Ivermectin and topical Permethrin, which eventually resulted in complete resolution. Interestingly, despite quarantining efforts, one of the patient's acquaintances and a healthcare worker acquired the symptoms of itching.

**Conclusion:**

This atypical presentation of Norwegian scabies emphasizes the need to include scabies in the differential diagnosis when HIV-infected patients present with crusted, generalized cutaneous lesions.

## Introduction

Norwegian (crusted) scabies is an opportunistic dermatological manifestation which is seen in HIV-infected individuals and which is probably acquired as a consequence of the immune system's inability to control the mites, thereby facilitating overwhelming reproduction [1]. There is a wide range of presentations of Norwegian scabies in people with HIV with lesions ranging from thick, crusted plaques to red papules to psoriasiform plaques to hyperkeratotic yellow papules [2,3]. The lesions in Norwegian scabies are classically distributed on the extremities, but are frequently found on the back, face, scalp and around the nail folds [4]. As Norwegian scabies is extremely infectious, early diagnosis is paramount to allow prompt therapeutic interventions and infection control. We report a case of a man being treated at a tertiary AIDS care centre in Chennai, India, who presented with severe Norwegian scabies infection with lesions distributed all over the body and which was initially misdiagnosed as a fungal skin infection.

## Case presentation

**Figure 1:**
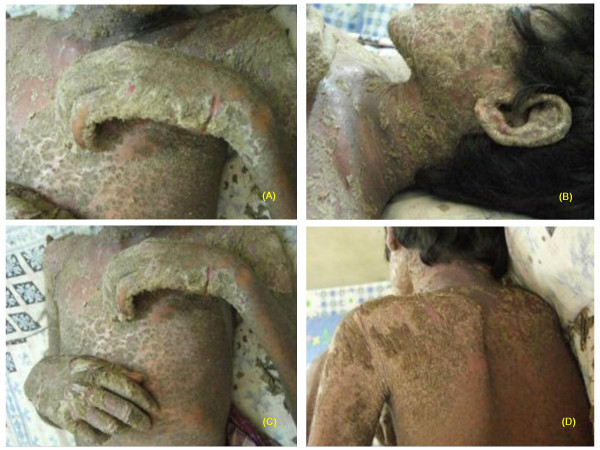
Extensive generalized, thick, hyperkeratotic, crusting, yellowish papule lesions distributed extensively over the limbs, ear lobes, face, trunk, shoulder blades and back (A-D) in a 16-year-old boy with HIV.

A 16-year-old man with HIV infection was admitted to the inpatient department of the YRG Centre for AIDS Research and Education (YRG CARE) with severe crusted cutaneous lesions all over the body. He had a history of skin lesions that had developed initially over the scalp and forehead, later spreading all over the body over the course of one month with no signs of itching. By the time of admission, the skin condition had worsened rapidly and there was extensive, generalized, thick, hyperkeratotic, crusting, yellowish papule lesions that eventually disseminated across the body including the face, ear lobes, shoulder blades and entire trunk, with squamous lesions not sparing any region (Figure 1). The patient presented with a temperature of 98.8°F and pulse rate of 80 beats a minute. The patient had a history of tuberculosis and had been on anti-tuberculosis therapy (ATT) for the past 3 years with poor adherence. Cardiovascular, respiratory and abdominal examinations were normal. Renal and liver function tests were also normal.

Laboratory investigations revealed a hemoglobin (Hb) level of 11.4 g/dl (normal values are in the range 12–17 g/dl), erythrocyte sedimentation rate (ESR) of 20 mm (normal range 0–14 mm), total thrombocyte count of 144 × 109/l (normal range 137–367 × 109/l), total leukocyte count of 18.2 × 109/l (normal range 3.9–9.4 × 109/l) and a total lymphocyte count (TLC) of 3.7 × 109/l (normal range 1.2–3.4 × 109/l). His absolute CD4 T-lymphocyte count (Beckman Coulter Inc., CA, USA) was 342 cells/μl (normal range 350–1411 cells/μl) and the CD4 T-lymphocyte percentage was 8%. His liver function test (LFT) revealed low alanine aminotransferase (ALT) at 28 IU/l (normal range 0–54 IU/l), normal total bilirubin of 0.4 mg/dl (normal range 0.4–2.3 mg/dl), direct bilirubin of 0.1 mg/dl (normal range 0.1–0.3 mg/dl) and a normal conjugate bilirubin of 0.3 mg/dl (normal range 0–1.5 mg/dl). His renal function tests revealed a low urine creatinine 0.5 mg/dl (normal range 0.9–1.3) and blood urea 11 mg/dl (normal range 9–33 mg/dl).

The differential diagnosis was initially either an adverse drug reaction, atopic dermatitis, dermatitis herpetiformis, psoriasis, ichthyosis, seborrheic dermatitis, erythroderma or Langerhans cell histiocytosis. However, following the suspicions of dermatologists of possible *Acarus scabiei *infestation, skin crusts were collected and mounted on 10% KOH preparation and observed under low- and high-power objectives. Numerous live and motile, adult *A. scabiei *mites that measured about 400 μm long and 300 μm wide were seen, which confirmed the diagnosis of Norwegian scabies. The patient was started with Ivermectin (6 mg) for 15 days and topical Permethrin cream with meticulous scrubbing and cleansing of the skin, which eventually resulted in complete resolution 4 weeks later. Intriguingly, in spite of quarantining efforts, one of the patient's acquaintances and a healthcare worker acquired the symptoms of itching, and had to be treated with topical Permethrin cream for a week. However, the diagnosis of scabies was not confirmed in either the acquaintance or the healthcare worker.

## Conclusion

Norwegian scabies is reported to be extremely infectious. This case report is of a man with severe crusted scabies with lesions not sparing any region in the body, and which was initially misdiagnosed as a fungal skin infection. This is a very unusual presentation of Norwegian scabies in an HIV-positive patient. The laboratory diagnosis of Norwegian scabies is simple, but clinical suspicion is required on the part of attending healthcare workers. Infrequently, scabies is mistakenly reported initially to be an adverse drug reaction, psoriasis, systemic lupus erythematosus or bullous pemphigoid [5-7]. A condition known as *scabies incognito *is reported to alter the presentation of lesions, obscuring the clinical diagnosis [4]. Clinicians must therefore be aware of the possible manifestations of scabies, including cases where the head and neck are involved. Uncomplicated scabies in adults is typically described as a skin condition with sparing of the head and neck region; the presence of lesions on the head and neck may therefore divert the clinician's suspicion to other skin problems as happened in our case. Owing to the extremely contagious nature of crusted scabies, as well as its potential for complete cure with appropriate therapy, a high degree of suspicion for this ailment should be maintained in people with HIV, even when the lesions do not have the classical appearance. In additional, it should be noted that effective measures should be taken to prevent nosocomial spread, as the infestation can also spread to healthcare workers [8,9]. In spite of being a common infectious condition widely seen among HIV-positive people, this case exemplifies the ever-escalating unusual clinical presentations seen in people with HIV/AIDS.

## Competing interests

The author(s) declare that they have no competing interests.

## Authors' contributions

NK, SS, SMT and BD were involved in the case directly and drafted part of the manuscript. RV, RS, PB and EMS were involved in diagnosis, literature review and helped draft the manuscript. All the authors read and approved the final manuscript.

## Consent

Written informed consent was obtained from the patient for publication of this case report and accompanying images. A copy of the written consent is available for review by the Editor-in-Chief of this journal.
